# How peptide/MHC presence affects the dynamics of the LC13 T-cell receptor

**DOI:** 10.1038/s41598-019-38788-0

**Published:** 2019-02-25

**Authors:** Jose Luis Dominguez, Bernhard Knapp

**Affiliations:** 1 Department of Basic Sciences, International University of Catalonia, Barcelona, Spain; 2 Department of Statistics, Protein Informatics Group, University of Oxford, Oxford, UK

## Abstract

The interaction between T-cell receptors (TCRs) of T-cells and potentially immunogenic peptides presented by MHCs of antigen presenting cells is one of the most important mechanisms of the adaptive human immune system. A large number of structural simulations of the TCR/peptide/MHC system have been carried out. However, to date no study has investigated the differences of the dynamics between free TCRs and pMHC bound TCRs on a large scale. Here we present a study totalling 37 100 ns investigating the LC13 TCR in its free form as well as in complex with HLA-B*08:01 and different peptides. Our results show that the dynamics of the bound and unbound LC13 TCR differ significantly. This is reflected in (a) expected results such as an increased flexibility and increased solvent accessible surface of the CDRs of unbound TCR simulations but also in (b) less expected results such as lower CDR distances and compactness as well as alteration in the hydrogen bond network around CDR3α of unbound TCR simulations. Our study further emphasises the structural flexibility of TCRs and confirms the importance of the CDR3 loops for the adoption to MHC.

## Introduction

Structural simulations are an important research method. Their performance and versatility have been consistently improved over the course of the years^1^ allowing these simulations to provide deep insights into interaction processes. Structural simulations have provided understanding of mechanisms that govern biology at a molecular level^2^ by simulating interactions between different acting agents at atomistic scale^3,4^, an insight that cannot be achieved at this resolution by experimental techniques such as crystallography or NMR. One of the main applications has been the observation of protein-substrate interactions, in order to determine how the presence of substrates alters the behaviour of the complexed protein and best discern how proteins executes their function. Examples include ligand binding to Muscarinic G-coupled receptors^5^, trypsin^6^, or kinases^7^.

**Figure 1:**
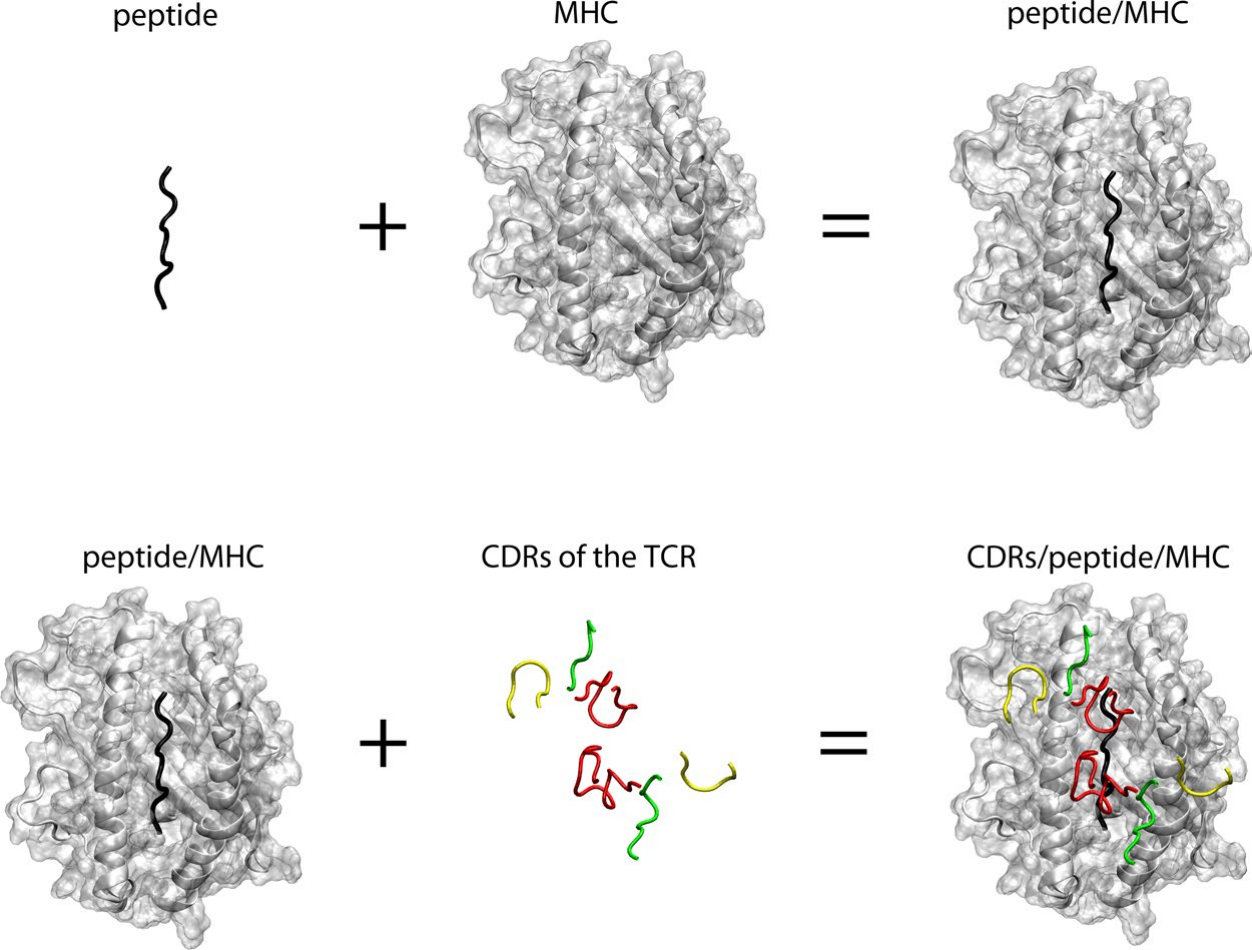
Top view on the TCR(CDR)/peptide/MHC interface. Black sticks: peptide; Grey cartoon and transparent surface: MHC; Green tubes: CDR1; Yellow tubes: CDR2; Red tubes: CDR3.

In this work we use structural simulations to investigate the effect of Major Histocompatibility Complex (MHC) binding to T-cell receptors (TCR). TCRs are cell surface receptors of T-cells that scan other cells for potentially immunogenic antigen fragments^8^. These fragments are presented to TCRs as elongated peptides in the binding groove of MHCs on the surface of antigen presenting cells^9,10^. When an immunogenic peptide/MHC (pMHC) combination is recognised by a TCR the T-cell is activated through a signalling cascade. However, the precise mechanism of very early T-cell activation is not known yet^8^: How does the signal travel from the pMHC interface (Fig. 1) via the complementarity determining regions (CDRs) through the TCR? A large number of computational studies have investigated this interaction (reviewed in^11^) including different peptide ligands^12–18^, peptide/MHC binding^19^ and detachment^20,21^, TCR/MHC interface interactions^22^, different TCR types^23^, different MHC types^24–26^, empty MHC binding grooves^27,28^, MHC plasticity^29^, MHC-tapasin interaction^30^, and effects of TCR binding on the MHC^31^. What has been missing so far is a large scale investigating of the effect of pMHC presence on the TCR dynamics. In this study we use structural simulations totalling 37 100 ns of simulation time to investigate the effect of peptide/HLA-B*08:01 MHC ligands on the dynamics of the LC13 TCR.

## Methods

### Structural basis

We downloaded the X-ray structure of the LC13 TCR in complex with the human MHC HLA-B*08:01 presenting the Epstein Barr Virus peptide FLRGRAYGL from the Protein Data Bank (PDB) (accession Id 1MI5)^32^. We chose this structure as it has been the subject of intensive investigation before^14,15,33^. The whole structure including the MHC α3-regions and β2-microglobulin as well as the constant regions of the TCR were used as we have recently shown that this is important for reliable conclusions^34^.

### Molecular simulation protocol

We simulated the TCR in its free form as well as in its TCRpMHC form. The structures were submerged into a dodecahedral simulation box filled with explicit SPC water allowing for a minimum distance of 1.2 nm between protein and box boundary. We added Na + and Cl− ions to achieve a neutral charge in the medium and reach a saline concentration of 0.15 M. The protonation state of the amino acids was automatically determined by Gromacs^35^. We first applied a steepest descent minimization, then warmed the system up to 310 K employing position restraints. Finally we carried out the molecular dynamics (MD) production runs using Gromacs 4^35^ and the GROMOS 53a6 force field^36^ using parameters derived from our previous study^37^.

Multiple Replicas (identical parameters but different seeds for initial velocities) per simulation are important for reproducible conclusions^19,20,22,38,39^. We performed 100 replicas of 100 ns each for the TCR system (referred to as “TCR”) as well as the wild-type TCRpMHC system (referred to as “TCRpMHC WT”).

In addition we used a set of 172 previously published^14^ TCRpMHC MD simulations of the same system. This data set contains all possible single point mutations of the nonamer FLRGRAYGL peptide (ALRGRAYGL, CLRGRAYGL, … FLRGRAYGY) in complex with HLA-B*08:01 and the LC13 TCR and the wildtype (172 = 19 × 9 + 1). The peptide mutants were created by SCWRL^40^ using the PeptX framework^41^. All 172 peptides are at least moderate binders^14^ and for each of the mutants an experimental immunogenicity value exists^32^ but we have shown before that there is no relevant difference in the TCR dynamics between more and less immunogenic recognition processes^14^. In this study we therefore treat all 171 mutant simulations as one group (“TCRpMHC MT”).

This led in total to a structural basis of 37100 ns (=100 × 100 TCR + 100 × 100 TCRpMHC WT + 171 × 100 TCRpMHC MT) for the current study.

### Descriptors of trajectory analysis

The trajectories were manually inspected using VMD^42^ and the vmdICE-plugin^43^. We calculated solvent accessible surface area (SASA) root mean square fluctuation (RMSF), radius of gyration, hydrogen bonds (H-bonds) and distances using the Gromacs modules gmx sasa, gmx rmsf, gmx gyrate, gmx hbond and gmx distance respectively. Results were imported into pymol/Matlab using gro2mat^44^ and the H-bond networks were created by pyHVis3D^45^.

### Comparison between TCRpMHC WT, TCRpMHC MT and unbound TCR simulations

We used three different types of measurements to quantify the pairwise magnitude of difference between descriptors of our three datasets. Firstly, the simple difference in mean value between two sets of simulations:

$$diff=\bar{X}-\bar{Y}$$

where X and Y are all the descriptor values of all time frames (100 ns) and all replicas of a specific descriptor (e.g. SASA or H-bonds). For example X could be TCRpMHC WT simulations and Y unbound TCR simulations. Secondly, we calculated Cohen’s d to quantify the effect size:

$$cohend=\frac{\bar{X}-\bar{Y}}{s}$$

where s is the standard deviation. Here a small effects size is up to 0.2, medium up to 0.5 and large above 0.8. Thirdly we computed the total variation difference (tvd) to quantify the difference in probability distribution overlap:

$$tvd\,({f}_{1},{f}_{2})=\,\frac{1}{2}\,\int |\,{f}_{1}(X)-{f}_{2}(Y)|dx$$

where ƒ_1_(X) is the normalized distribution of X and ƒ_2_(Y) the normalized distribution of Y. Based on this calculation the tvd ranges from 0 to 1 where 0 represents perfect overlap of the distributions and a 1 represents no overlap. The tvd is always positive.

## Results

We have analysed a total of 371 simulations of 100 ns each separated into three groups of 100 TCRpMHC WT simulations, 100 TCR simulations, and 171 TCRpMHC MT simulations.

### Root Mean Square Fluctuation

**Figure 2:**
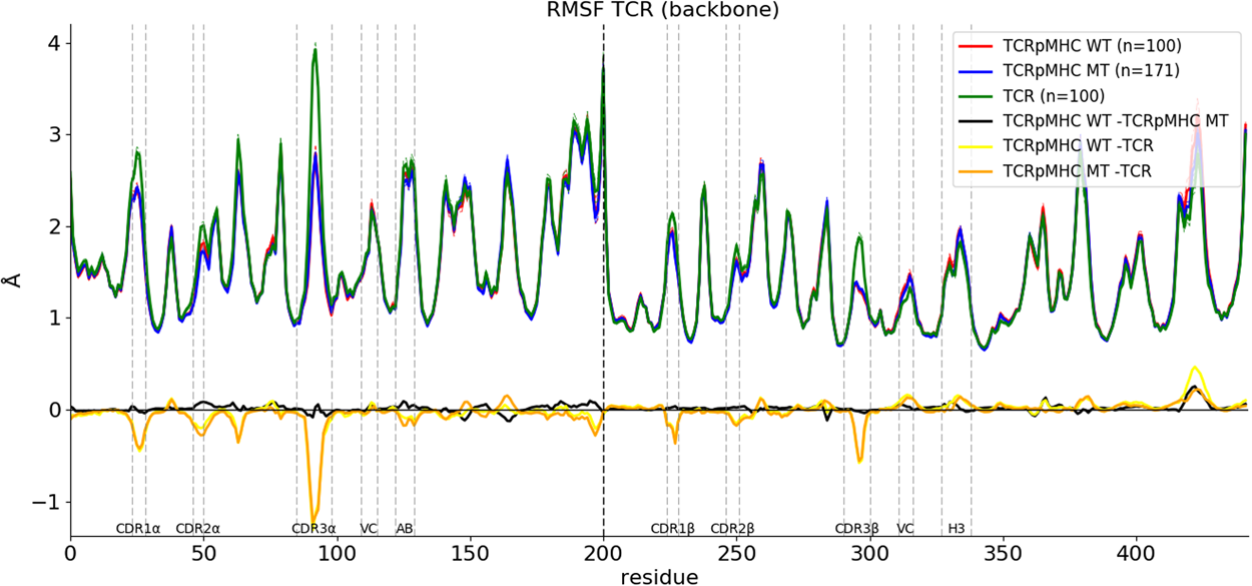
Mean RMSF values for the TCRpMHC WT (red), TCRpMHC MT (blue) and unbound TCR (green) simulations. Thin dashed lines of the same colour indicate +/− standard error of mean (very small as n is high). For easier interpretation the differences between the mean values are shown at the bottom in black, yellow, and orange. The thick vertical dashed line indicates the border between the TCR alpha and beta chain.

Firstly, we analysed the mean RMSF of each of the three sets to investigate the flexibility/stability of different TCR regions (Fig. 2). In this analysis higher values indicate more flexibility while lower values indicate lower flexibility. Shifts in RMSF values when comparing the different sets indicate which regions are particularly affected by the complex formation. The results show that the presence of pMHC reduces the flexibility of all six CDR regions but has surprisingly little effect elsewhere in the TCR. Especially the linking regions between the variable and constant domain (VC)^46^ and the previously described AB-linker^47^ and H3 region^48^ are not affected. The results also show that there are only very little differences between TCRpMHC WT and TCRpMHC MT simulations (black line in Fig. 2). It seems that binding of different mutant ligands does not alter the binding interface strongly.

### Distances between CDRs

**Figure 3:**
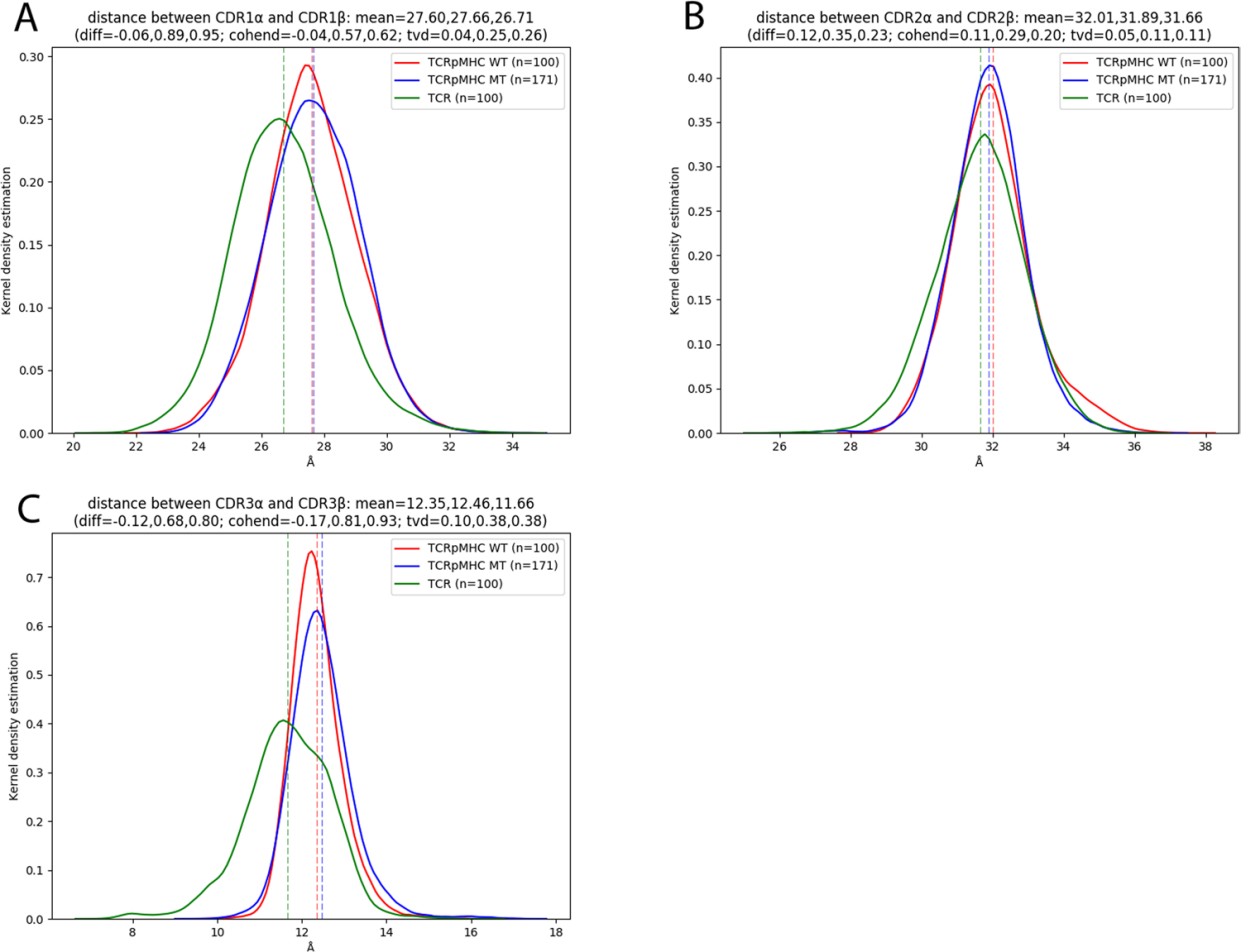
Distributions of the distances between the CDR loops for TCRpMHC, unbound TCR, and TCRpMHC mutants. Top left: CDR1. Top right: CDR2. Bottom: CDR3.

Next we investigated if the pMHC ligand causes an opening or closing of the TCR interface. The most important contact areas of TCR are the CDRs, where the two CDR3s are positioned centrally over the peptide and CDR1/2 bind over the MHC helices (Fig. 1). We quantified the distance between each pair the CDR loops (Fig. 3). It can be seen that the unbound TCR has a decreased distance between the CDR1s as compared to TCRpMHC simulations and that there is no relevant difference between TCRpMHC WT and TCRpMHC MT simulations (Fig. 3A). In the case of CDR2 only a small difference is present between the three sets (Fig. 3B) while the strongest effect can be observed for CDR3 (Fig. 3C). TCR simulations have a strongly decreased distance indicating a closing of the TCR interface in the absence of a pMHC ligand.

### Hydrogen Bonds

**Figure 4:**
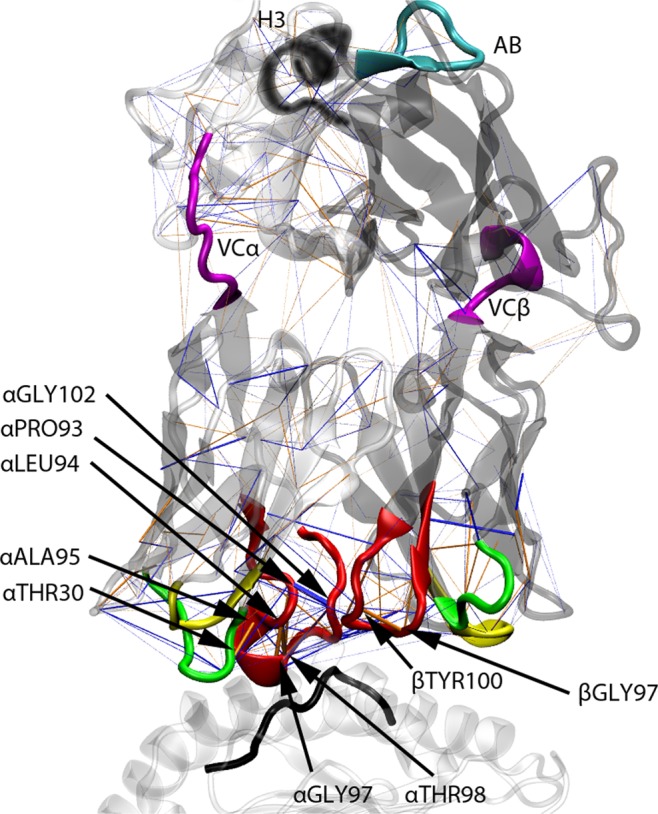
3D visualisation of the H-bond network difference between TCR and TCRpMHC WT simulations using pyHVis3D^45^. Orange lines indicate more H-bonds in TCRpMHC simulations while blue lines indicate more H-Bonds in TCR simulations. The thickness of the line is proportional to the magnitude of difference. Black tube: peptide; White transparent cartoon on the bottom: MHC; White transparent cartoon top left: TCR α-chain; Grey transparent cartoon top right: TCR β-chain. Green tubes: CDR1; Yellow tubes: CDR2; Red tubes: CDR3; Magenta tubes: linking regions between variable and constant regions of the TCR; Cyan tube: AB-loop; Black tube: H3 region.

To further investigate the tightness of the TCR interface we analysed the number of H-bonds between the TCR α and β chain. The average number of H-bonds over all time frames and replicas is highly similar, yielding 15.28 for TCRpMHC simulations, 15.79 for unbound TCR simulations and 15.05 for TCRpMHC mutant simulations (Fig. S1). This suggests that the core structure of the TCR remains mostly unchanged upon pMHC binding. This observation is further confirmed by a 3D visualisation of the differences in the H-bond network (Fig. 4) which shows that apart from TCR/MHC interface near residues little difference is visible. The largest differences can be observed in an on average 0.26 H-bonds increased presence between TCRαPRO93 and TCRαGLY102 (both part of CDR3α) for TCR simulations as compared to TCRpMHC simulations. In contrast TCR simulations show a 0.21 decreased presence between TCRαLEU94 and TCRαTHR98 (both part of CDR3α) as well as a 0.21 decreased presence between TCRαLEU94 and TCRαGLY97 (also both part of CDR3α). The fourth and fifth largest differences are decreases of 0.19 H-bonds between TCRβGLY97 and TCRβTYR100 (both part of CDR3β) and 0.18 H-bonds for TCRαTHR30 and TCRαALAα95 (between CDR1α and CDR3α). More numerical details are available in the supplementary material (Table S 1).

### Solvent Accessible Surface Area of CDRs

**Figure 5:**
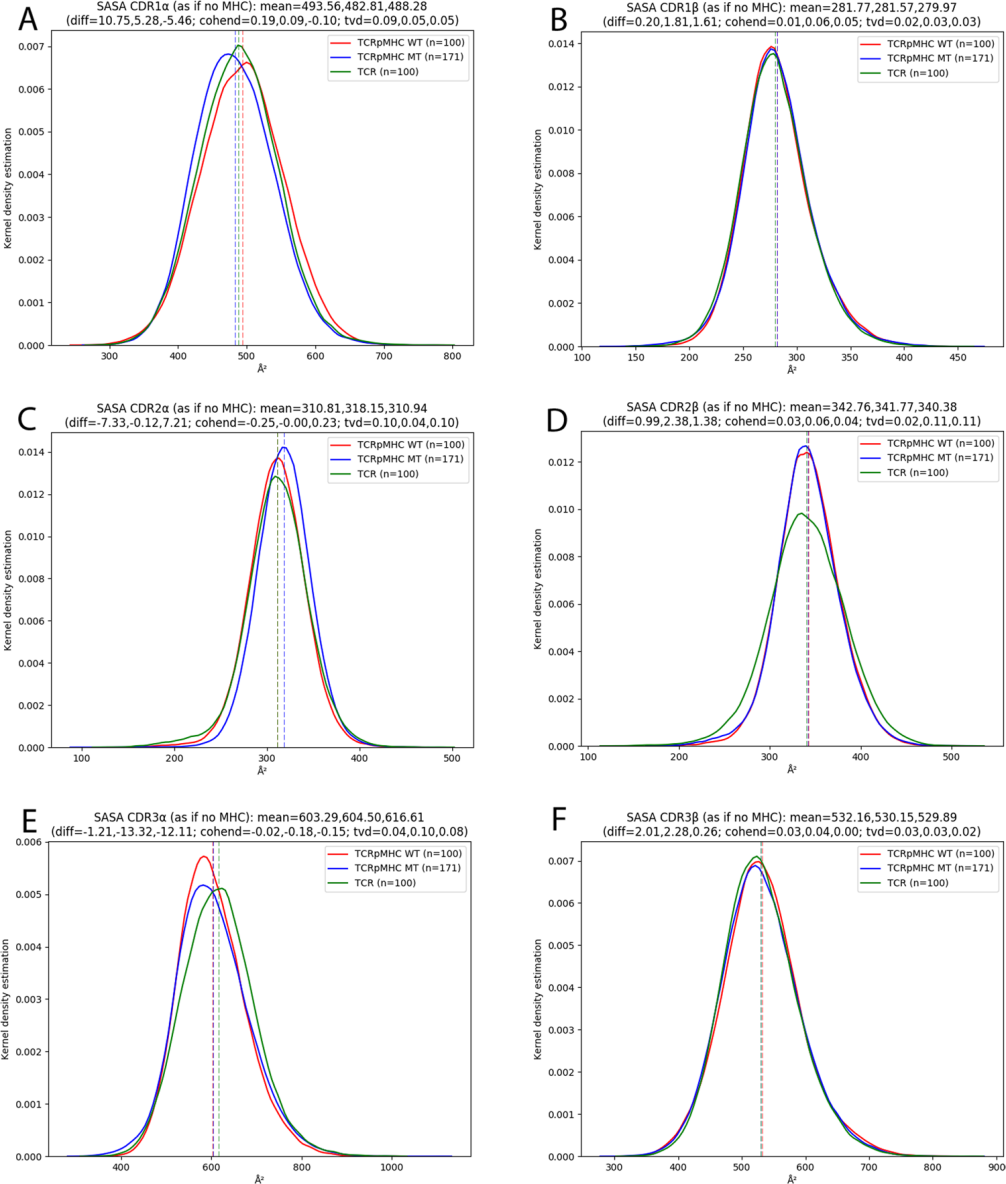
Theoretical SASA of the six CDR loops not taking into account the presence of the MHC for the SASA calculation. The original SASA values are shown in Fig. S2. (**A**) CDR1α. (**B**) CDR1β. (**C**) CDR2α. (**D**) CDR2β. (**E**) CDR3α. (**F**) CDR3β.

We also analysed the SASA of the six CDR loops. The SASA quantifies the amount of exposure of a given region to the solvent medium. As expected the binding of a pMHC ligand causes a strong (up to 50%) reduction in the SASA of the CDRs (Fig. S2). However, there are two more interesting questions to be asked about the SASA: Firstly, do TCRpMHC MT simulations cause different SASA patterns in the CDRs than the TCRpMHC WT simulations? Although the mean SASA values are almost identical (Fig. S2) the shape of the distribution for CDR1α changes: wild-type simulations have a broader distribution while mutant simulations have a sharper distribution (11% change in overlay Fig. S2A). For the rest of the CDRs no difference is present. Secondly, is the topology of the CDR surface altered? Working with simulations also allows us to measure the hypothetical SASA of each of the CDRs as if the pMHC ligand was not present (even if pMHC was present during the simulation). This answers the question if the topography of the CDR surfaces is altered by the pMHC binding. Figure 5 shows that only slight changes are present: For example CDR1α has a slightly lower SASA for TCRpMHC MT simulations than for TCRpMHC WT simulations (Fig. 5A). In contrast CDR2α has a slightly increased SASA for TCRpMHC MT simulations than for TCR or TCRpMHC WT simulations (Fig. 5C). CDR2β has a slightly broader distribution of SASA values for unbound TCR simulations while the mean values are almost identical (Fig. 5D). CDR3α has an increased SASA for unbound TCR simulations as compared to TCRpMHC simulations.

### Radius of Gyration of CDRs

**Figure 6:**
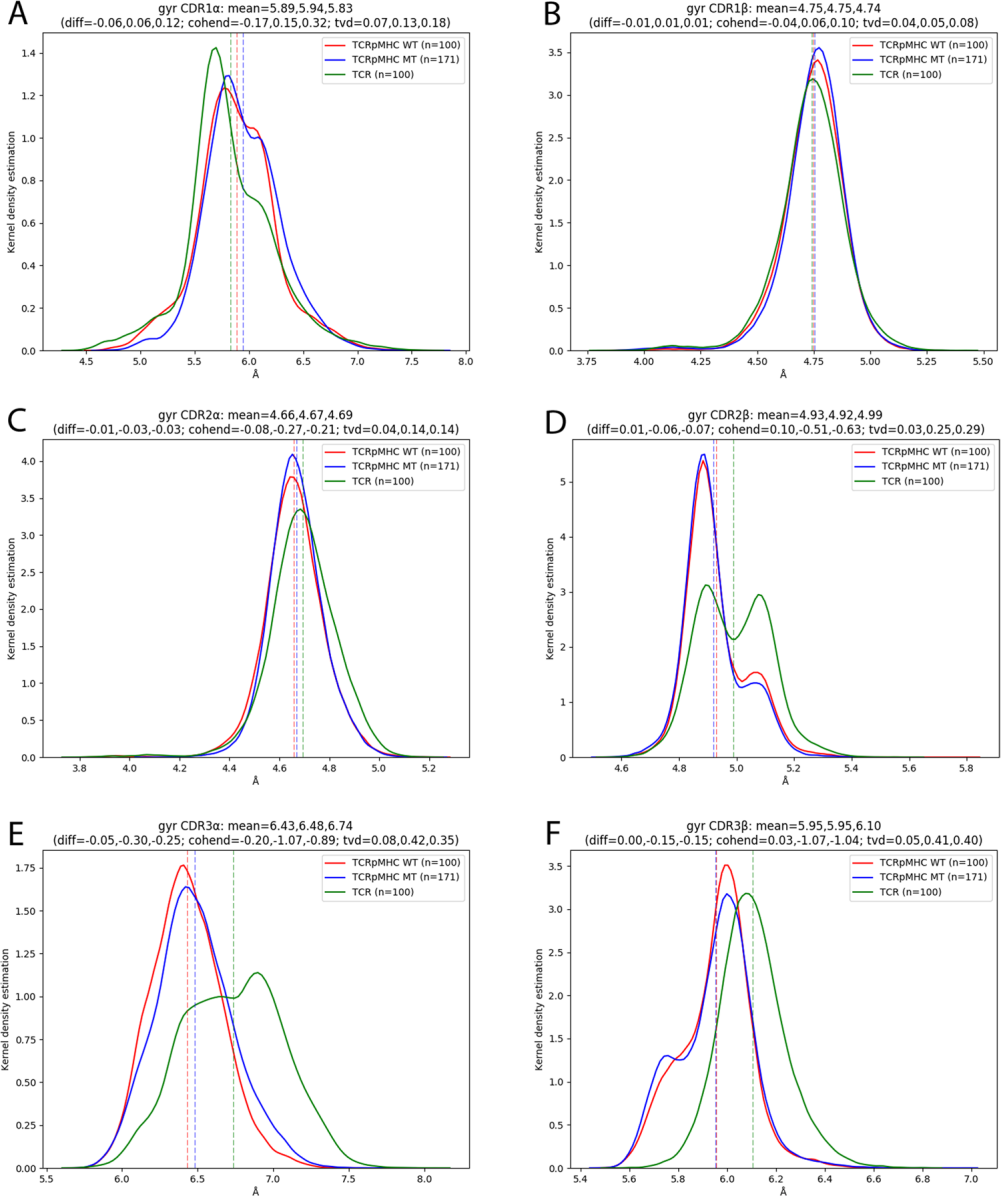
Radius of gyration of the six CDR loops. (**A**) CDR1α. (**B**) CDR1β. Cleft: CDR2α. Middle Right: CDR2β. Bottom left: CDR3α. Bottom Right: CDR3β.

As the SASA analysis indicated slight changes in the CDR shape we further investigated this change using the radius of gyration (rgyr). The rgyr is an approximation of the compactness of a structure: A high rgyr indicates a more extended structure while a low rgyr indicates a more compact structure. We measured the rgyr of the six CDR regions (Fig. 6) in order to investigate if pMHC presence affects the compactness of these regions.

For CDR1α TCR simulations prefer a more compact configuration than TCRpMHC simulations. This is visible in the distribution as a more populated left peak in TCR simulations as compared to a more populated right peak in TCRpMHC simulations (tvd = 0.13 and 0.18 Fig. 6A). For CDR1β no relevant difference is visible (Fig. 6B) while for CDR2α TCR simulations show a marginally more extended configuration (Fig. 6C). For CDR2β simulations show bi-modal distribution while the more extended state is more populated in TCR simulations then in TCRpMHC simulations (Fig. 6D). The strongest difference between TCR and TCRpMHC simulations is however observable in the CDR3s. TCR simulations tend to have more extended conformations for CDR3α as well as CDR3β than TCRpMHC simulations (Fig. 6E,F). TCRpMHC WT and TCRpMHC MT simulations do not show relevant differences from each other.

### Further areas of interest

**Figure 7:**
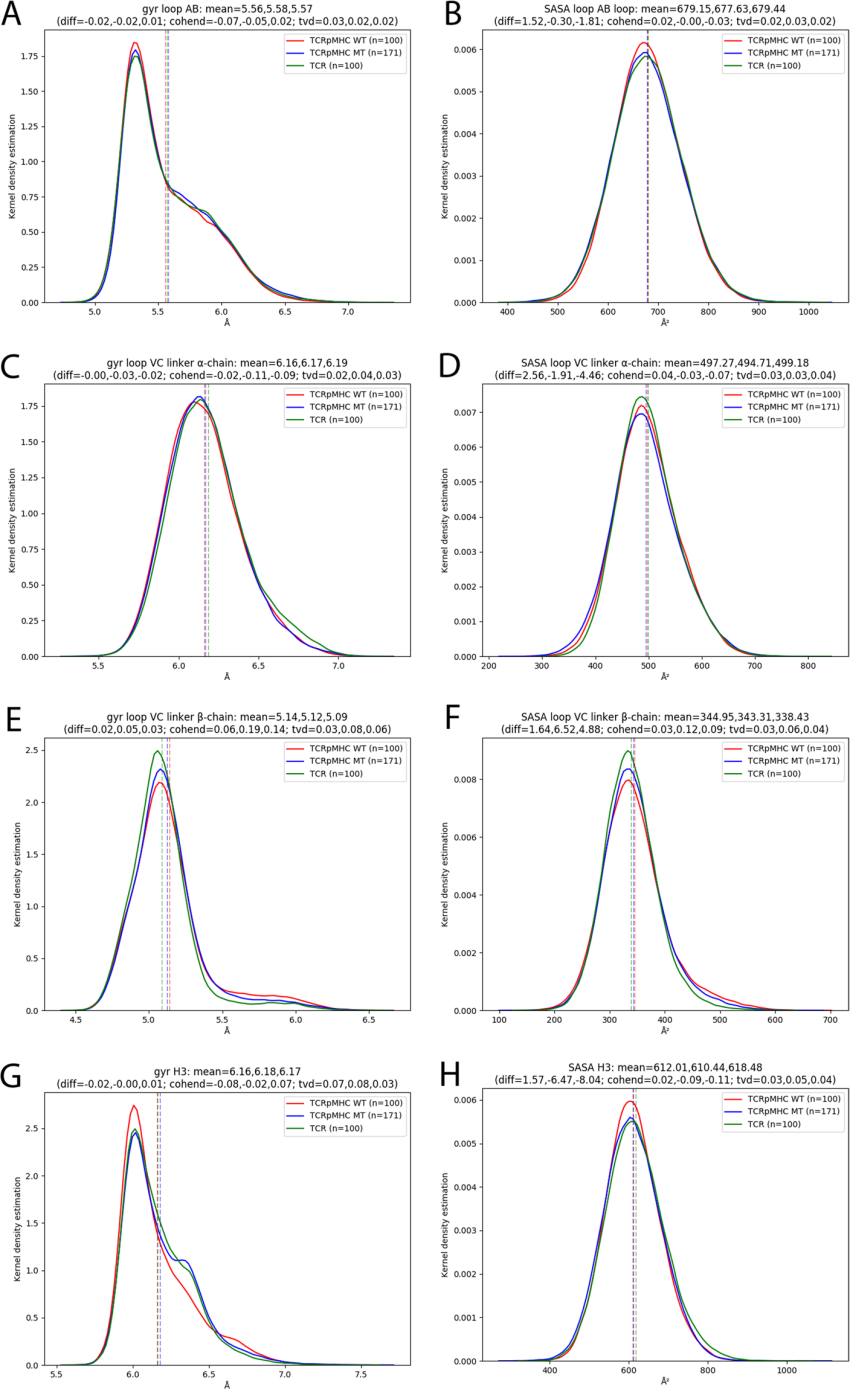
Analysis of non-CDR loops of interest. The analysis includes linking regions between the variable and constant domain (VC)^46^ and the previously described AB-linker^47^ and H3 region^48^. (**A**) Radius of gyration of the AB-loop, (**B**) SASA of the AB-loop, (**C**) Radius of gyration of the VCalinker, (**D**) SASA of the VCa-linker, (**E**) Radius of gyration of the VCb-linker, (**F**) SASA of the VCb-linker, (**G**) Radius of gyration of the H3 region, (**H**) SASA of the H3 region.

In addition to the CDRs we also investigated the details of four more regions that have been reported to be import in TCR signalling: the AB-loop^47^, the H3 region^48^ as well as the two regions linking the variable to the constant domains (VC) of the TCR (see Fig. 4 for a visualisation). The RMSF (Fig. 2) and SASA (Fig. 7) do not show relevant differences in these four regions between our three groups. Only the radius of gyration of the VCβ is slightly lower in TCR simulations than in TCRpMHC simulations indicating a slightly more compact conformation (Fig. 7E). This lack of differences in important hinge regions agrees with the global impression of the H-bond network (Fig. 4) and RMSF (Fig. 2) that shows no global changes or relevant differences in the TCR that are located further away from the MHC binding site.

### Separate analysis of peptide mutants

In the current study we treated all 171 peptide mutants as one group (entitled MT). To provide further insight into the relation of individual peptide properties with the molecular descriptors of this study we have added a Pearson correlation analysis between molecular weight, hydrophobicity, and experimentally measured immunogenicity of the peptides on the one hand and the molecular descriptors of this study on the other hand to the supplementary material of this study (Table S 2). It can be seen that there is no relevant correlation within these 75 tests performed. The highest correlation found is 0.211 between the molecular weight of the peptide mutants and the SASA of CDR1ß. This lack of findings agrees with our previous study^14^ that showed that simulation derived descriptors are not sufficient to predict experimentally measured immunogenicity.

## Discussion

### A large number of replicas is needed for reliable conclusions

We haven recently shown that multiple replicas of the same molecular simulation are important for reproducible conclusions^38^. Therefore we analysed a total of 37.1 µs of MD simulation trajectories of a single TCRpMHC system to obtain a detailed and reliable characterisation of the dynamics of the LC13 TCR in its unbound as well as pMHC bound form. To our knowledge this is the largest MD study of TCRpMHC interactions. The large number of simulations per group allowed us to draw reliable conclusions as single simulations of finite length are often stochastic in terms of which part of the solution space they sample^19,49^ and convergence of simulations is questionable^50,51^. This behaviour is mainly caused by a rugged solution landscape^52^ and minimal discrepancies in floating point precision, number of processors, type of processor, compiling options, random number generators, and/or dynamic load balancing^49^. Therefore a large number of replicas/simulations per group benefits the reproducibility of results. This was for example shown for peptide/MHC detachments^20^, TCR/pMHC interactions^22^, and peptide/MHC binding affinity predictions^19^, but also for different systems such as HIV protease inhibitors^39^.

### Recovery of expected results

If two proteins are bound to each other it can be expected that the interface area is affected in two ways^53^. Firstly, there would be significantly less water around the binding interface than in the unbound state. In our study the interface consists of the genetically and structurally variable six CDRs loops. Consequently we found that the CDRs of unbound TCR simulations have on average 158.8% of the SASA of TCRpMHC simulations. Secondly, the interface residues would have a lower flexibility if bound to another protein. Also this feature of the binding interface was recovered. While the average CDR RMSF of TCRpMHC-WT simulations was 1.612 Å, the RMSF value for unbound TCR simulations was 1.813 Å (112.5%).

### Non-expected results

While the results above are an interesting quantification of differences between the bound and unbound state the general tendency of these results could have been known before performing the simulations. However, in our dataset we also found differences between TCRpMHC and unbound TCR simulations that could not have been anticipated before: the intra-CDR distance is smaller for CDR 1 and CDR3 when comparing unbound TCR simulations with TCRpMHC simulation: 26.71 Å vs 27.60 Å for CDR1 and vs 11.66 Å vs 12.35 Å for CDR3. Also the compactness of the CDR loops is influenced by pMHC presence. CDR1α shows a lower radius of gyration for TCR simulations while CDR2β and CDR3α show a higher and bimodally distributed radius of gyration for TCR simulations. For CDR3β, unbound TCR simulations show a higher radius of gyration while the bimodal distribution is present only for TCRpMHC simulations. These findings are further emphasised by the analysis of the H-bond network that shows that mainly CDR3α and its interaction with CDR3β is influenced by pMHC presence. Taken together, this indicates an adjustment of the TCR to the pMHC ligand by an opening of the TCR binding site which is in agreement with previous studies that suggest mechanical forces as part of TCR signalling^46,54^.

### pMHC presence mainly affects the CDR loops of the TCR

In our analysis we found that the TCR dynamics between unbound TCR and TCRpMHC simulations mainly differ in the CDR regions which are directly adjacent to the pMHC binding site. Here especially the CDR3 loops are affected. These findings are consistent with significant differences in the CDR X-ray structures of bound and unbound TCRs^32,55^ as well as the idea that pMHC binding might occur in a two-step process where the second step is more specific and affects the CDR3 regions^56^. Our results do not reproduce previously suggested changes in the VC linkers^46^, AB-loop^32^, H3 region^48^, and quaternary structure of the TCR^46^. This discrepancy might be explained by the limited time scale of MD simulations and/or TCR and MHC allele specific behaviour.

### Most changes of the LC13 TCR are not conserved for the A6 TCR

In this study we have shown a number of dynamical effects that pMHC ligandation has on the LC 13TCR. TCR triggering is known to be highly dependent on all three interaction partners (TCR, peptide and MHC). Nevertheless the question arises if our LC13 TCR results are conserved for other TCRs. For this purpose we additionally performed 10 replicas of 100 ns each of the A6-TCR/LLFGYPVYV/HLA-A*02:01 (PDB accession code *1AO7*) as well as 10 replicas of 100 ns each of the A6 TCR in its unbound form. We analysed the A6 TCR following the same methodology as for the LC13 TCR. In Table S 1 we compare the results for the two TCRs. Agreement between the TCRs can only be found in expected properties as a larger SASA and higher RMSF of CDRs of unbound TCRs. Also the distance between the CDR2 loops is higher in pMHC bound TCRs than in unbound TCRs for both the LC13 TCR and the A6 TCR. This effect is almost 4 times as high in the A6 TCR as compared to the LC13 TCR making also this an unlikely conserved property of TCR activation.

The non-conserved effects found in this study agree well with previous experimental research that could not find conserved patterns between different TCRs. For example for the LC13 TCR the X-ray structures of the bound and unbound TCR show differences in the AB-linker^47^. To our knowledge these results could not be reproduced for any other TCR. For the B4.2.3 TCR structural effects were demonstrated in the H3 region when comparing the bound and unbound TCR^48^ but also in this case inspection of the 10 available TCRpMHC structures for which also an unbound TCR structure exists did not show consistent differences in the H3 backbone configurations or side-chain orientations^48^.

As ligandation effects on the TCR are highly dependent on all three interaction partners it is also not expected that effects would be conserved between MHC class I and class II as these two complexes despite sharing the same overall fold differ markedly from each other. This includes the open MHC binding groove for class II as well as that MHC I consists of one long α-chain that is in TCR contact while MHC II consists of an α and a β chain that are in TCR contact^9^.

### Biological view

Despite many years of research the drivers of MHC restriction of TCRs^57^ and mechanistic details of TCR triggering by pMHC remain challenges^8^. Three major hypotheses of TCR triggering including aggregation, segregation, and conformational change exist^8^. All three hypotheses include, or at least allow, the possibility of structural effects or subtle rearrangements within the TCR. There are five lines of evidence for the importantance of structural effects in TCR triggering (reviewed in^8^): (1) A certain degree of mechanical force on the TCRs during binding to the peptide/MHC complex is inevitable^58^, (2) the TCR is in physical contact with CD3 allowing the possibility of signal transduction into the cell^59^, (3) if the ligands are anchored to a surface T cell activation is optimal^60^, (4) elongation of peptide/MHC can inhibit TCR triggering^61^, (5) mechanical forces on the TCR enhance TCR triggering^46,54,62^. However, despite the overall similar shape of different TCRs a common structural pattern of how a TCR transmits activation signals has not yet been found. In this study we have provided an in-depth analysis of the dynamics of the LC13 TCR at an atomistic level highlighting a number of dynamical differences between the bound and unbound TCR dynamics. However, much like the differences found in X-ray structures, these differences in dynamics might be TCR and MHC allele specific and are no general patterns of TCR triggering.

## Conclusion

We have, to our knowledge, presented the largest study investigating the dynamics of a bound and unbound TCR. Overall, our results suggest a flexible interface of the TCR that reacts to pMHC binding. Our study confirms the importance of the CDR3 loops in pMHC recognition and supports the hypothesis^46,54^ that mechanical forces might play a role in TCR activation.

## Supplementary information

Supplementary Material

## Data Availability

The datasets generated during and/or analysed during the current study are available from the corresponding author on reasonable request.
