# Supplementary material for: How peptide/MHC presence affects the dynamics of the LC13 T-cell receptor
Source: Sci Rep. 2019 Feb 25;9:2638. doi: 10.1038/s41598-019-38788-0 (PMC6389892; doi:10.1038/s41598-019-38788-0)
Supplement: Supplementary file 1 — Supplementary Material [file 41598_2019_38788_MOESM1_ESM.pdf]

Supplementary Material

How peptide/MHC presence affects the dynamics of the LC13 T-cell receptor

Jose Luis Dominguez Meijide[1], Bernhard Knapp[1,2,*]

[1] Department of Basic Sciences, International University of Catalonia, Spain

[2] Department of Statistics, Protein Informatics Group, University of Oxford

* science.bernhard.knapp@gmail.com

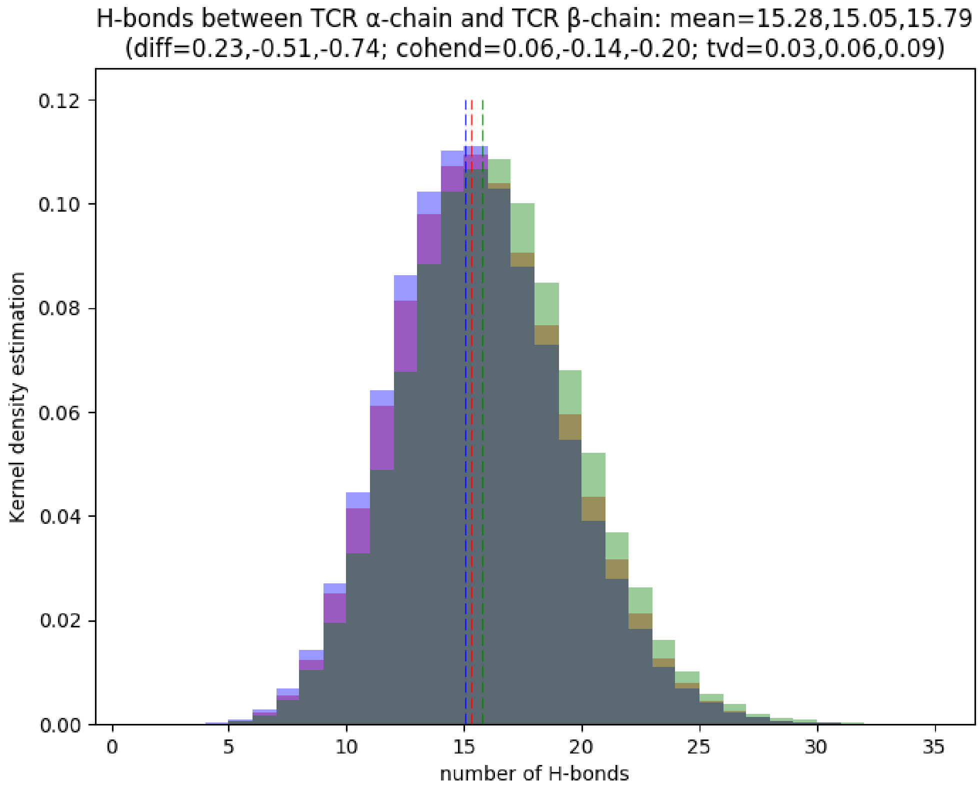

Figure S 1: Histogram of the number of Hydrogen Bonds in the different MD trajectory sets. The dashed lines represent the mean values for TCRpMHC (red), TCRpMHC mutants complex (blue) and unbound TCR (green).

```
------- 10 MOST FREQUENT H-BOND DIFFERENCES -------
H-bond from PRO93(D) to GLY102(D) (117.240 124.680 59.000; 121.610 123.350 59.230)
presence 0.26
H-bond from LEU94(D) to THR98(D) (118.900 126.270 55.960; 121.790 132.060 55.470)
presence -0.21
H-bond from LEU94(D) to GLY97(D) (118.900 126.270 55.960; 121.510 129.590 52.570)
presence -0.21
H-bond from GLY97(E) to TYR100(E) (128.280 117.300 59.370; 123.210 116.140 58.040)
presence -0.19
H-bond from THR30(D) to ALA95(D) (117.230 126.140 47.980; 117.100 129.610 55.530)
presence -0.18
H-bond from ASN51(E) to GLU52(E) (135.420 119.680 65.550; 137.060 122.030 68.130)
presence -0.14
H-bond from PRO93(D) to THR98(D) (117.240 124.680 59.000; 121.790 132.060 55.470)
presence -0.13
H-bond from TRP34(E) to SER75(E) (123.850 122.440 71.940; 131.740 118.720 75.030)
presence 0.13
H-bond from SER99(D) to GLY102(D) (122.010 129.160 57.940; 121.610 123.350 59.230)
presence -0.12
H-bond from TYR100(D) to GLN106(E) (124.140 125.930 57.950; 121.090 115.270 64.020)
presence -0.11
```

Table S 1: 10 highest H-bond frequency differences between TCR and TCRpMHC-WT simulations.

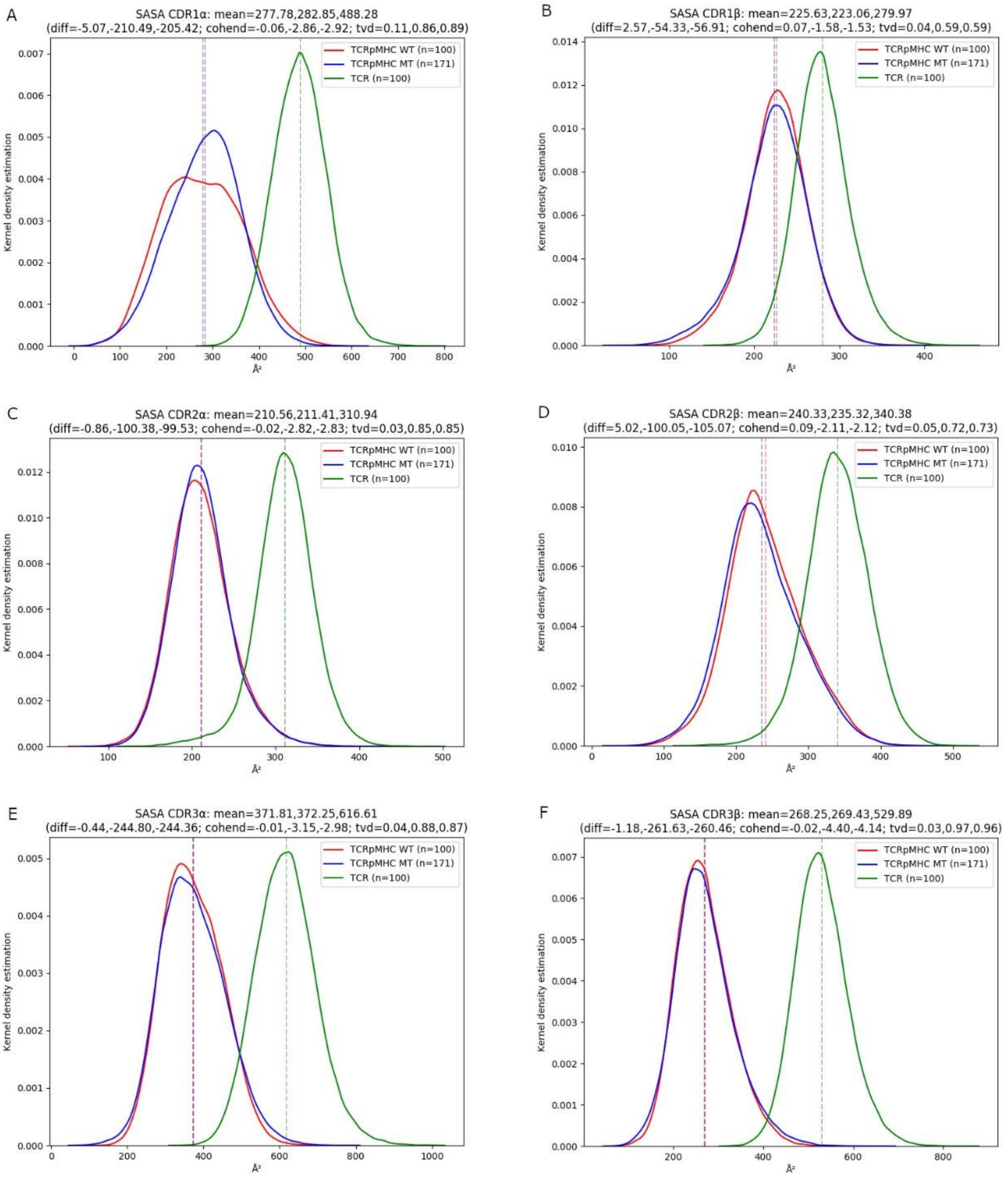

Figure S 2: SASA of the six CDR loops. A: CDR1α. B: CDR1β. C: CDR2α. D: CDR2β. E: CDR3α. F: CDR3β.

Pearson correlation coefficients of mutants

		cc	p-value
distance between CDR1a and CDR1ß	molecular weight	-0,018	0,814
distance between CDR1a and CDR1ß	hydrophobicity	-0,021	0,788
distance between CDR1a and CDR1ß	immunogenicity	0,064	0,407
distance between CDR2a and CDR2ß	molecular weight	0,076	0,324
**distance between CDR2a and CDR2ß**	**hydrophobicity**	**-0,150**	**0,049**
distance between CDR2a and CDR2ß	immunogenicity	0,048	0,533
**distance between CDR3a and CDR3ß**	**molecular weight**	**-0,197**	**0,010**
distance between CDR3a and CDR3ß	hydrophobicity	0,055	0,471
distance between CDR3a and CDR3ß	immunogenicity	0,018	0,810
RMSF TCR (backbone)	molecular weight	0,026	0,740
RMSF TCR (backbone)	hydrophobicity	-0,147	0,054
RMSF TCR (backbone)	immunogenicity	0,108	0,160
H-bonds between TCR a-chain and TCR ß-chain	molecular weight	0,054	0,483
H-bonds between TCR a-chain and TCR ß-chain	hydrophobicity	0,060	0,432
H-bonds between TCR a-chain and TCR ß-chain	immunogenicity	0,008	0,921
SASA loop AB loop	molecular weight	-0,079	0,303
SASA loop AB loop	hydrophobicity	0,009	0,908
SASA loop AB loop	immunogenicity	0,094	0,222
SASA loop VC linker a-chain	molecular weight	0,045	0,555
SASA loop VC linker a-chain	hydrophobicity	-0,011	0,888
SASA loop VC linker a-chain	immunogenicity	0,025	0,742
SASA loop VC linker ß-chain	molecular weight	-0,100	0,192
SASA loop VC linker ß-chain	hydrophobicity	-0,065	0,400
SASA loop VC linker ß-chain	immunogenicity	0,057	0,457
SASA H3	molecular weight	-0,081	0,292
SASA H3	hydrophobicity	0,056	0,465
SASA H3	immunogenicity	0,008	0,917
SASA CDR1a	molecular weight	-0,010	0,896
SASA CDR1a	hydrophobicity	-0,004	0,956
SASA CDR1a	immunogenicity	0,049	0,527
SASA CDR2a	molecular weight	-0,051	0,507
SASA CDR2a	hydrophobicity	-0,137	0,073
SASA CDR2a	immunogenicity	0,094	0,221
SASA CDR3a	molecular weight	-0,011	0,885
SASA CDR3a	hydrophobicity	-0,064	0,407
SASA CDR3a	immunogenicity	0,018	0,816
**SASA CDR1ß**	**molecular weight**	**0,211**	**0,006**
SASA CDR1ß	hydrophobicity	0,039	0,615
SASA CDR1ß	immunogenicity	-0,050	0,511
SASA CDR2ß	molecular weight	0,093	0,225
SASA CDR2ß	hydrophobicity	-0,049	0,525
SASA CDR2ß	immunogenicity	-0,054	0,481
SASA CDR3ß	molecular weight	0,036	0,641
SASA CDR3ß	hydrophobicity	0,063	0,414
SASA CDR3ß	immunogenicity	-0,017	0,829
gyr loop AB	molecular weight	-0,021	0,783
gyr loop AB	hydrophobicity	0,012	0,877
gyr loop AB	immunogenicity	0,026	0,732
gyr loop VC linker a-chain	molecular weight	0,049	0,525
gyr loop VC linker a-chain	hydrophobicity	-0,020	0,797
gyr loop VC linker a-chain	immunogenicity	0,104	0,175

**gyr loop VC linker ß-chain**	**molecular weight**	**-0,169**	**0,027**
gyr loop VC linker ß-chain	hydrophobicity	-0,025	0,743
gyr loop VC linker ß-chain	immunogenicity	0,040	0,605
**gyr H3**	**molecular weight**	**-0,180**	**0,018**
gyr H3	hydrophobicity	0,070	0,364
gyr H3	immunogenicity	0,072	0,345
gyr CDR1a	molecular weight	0,065	0,400
gyr CDR1a	hydrophobicity	0,050	0,517
gyr CDR1a	immunogenicity	-0,139	0,070
gyr CDR2a	molecular weight	-0,047	0,540
gyr CDR2a	hydrophobicity	0,067	0,382
gyr CDR2a	immunogenicity	0,000	0,999
**gyr CDR3a**	**molecular weight**	**0,200**	**0,009**
gyr CDR3a	hydrophobicity	-0,010	0,894
gyr CDR3a	immunogenicity	-0,074	0,335
gyr CDR1ß	molecular weight	0,023	0,764
gyr CDR1ß	hydrophobicity	0,058	0,447
gyr CDR1ß	immunogenicity	-0,043	0,574
gyr CDR2ß	molecular weight	0,039	0,610
gyr CDR2ß	hydrophobicity	0,096	0,209
gyr CDR2ß	immunogenicity	-0,069	0,370
gyr CDR3ß	molecular weight	0,123	0,107
gyr CDR3ß	hydrophobicity	-0,061	0,425
gyr CDR3ß	immunogenicity	-0,090	0,239

Table S 2: Correlation analysis between peptide mutant properties (molecular weight, hydrophobicity, immunogenicity) and descriptors obtained from our simulations. Correlations with a p-values < 0.05 are highlighted in yellow.

distance	LC13	A6	LC13	A6	LC13	A6	LC13	A6
	difference: TCRpMHC minus TCR		permutation test percentile		permutation test binary		only significant differences	
dist_bb_CDR1	0,089	0,013	1,000	0,659	1	0	0,089	0,000
dist_bb_CDR2	0,035	0,139	0,994	1,000	1	1	**0,035**	**0,139**
dist_bb_CDR3	0,068	-0,031	1,000	0,095	1	0	0,068	0,000
gyrCDR1a	0,006	-0,002	0,922	0,458	0	0	0,000	0,000
gyrCDR1b	0,001	0,005	0,825	0,995	0	1	0,000	0,005
gyrCDR2a	-0,003	0,012	0,000	1,000	1	1	-0,003	0,012
gyrCDR2b	-0,006	0,008	0,000	0,970	1	0	-0,006	0,000
gyrCDR3a	-0,030	0,029	0,000	1,000	1	1	-0,030	0,029
gyrCDR3b	-0,015	-0,004	0,000	0,289	1	0	-0,015	0,000
sasaCDR1a noMHC	0,053	0,566	0,829	1,000	0	1	0,000	0,566
sasaCDR1b noMHC	0,018	-0,201	0,801	0,008	0	1	0,000	-0,201
sasaCDR2a noMHC	-0,001	0,210	0,453	0,985	0	1	0,000	0,210
sasaCDR2b noMHC	0,024	-0,062	0,853	0,138	0	0	0,000	0,000
sasaCDR3a noMHC	-0,133	0,574	0,018	1,000	1	1	-0,133	0,574
sasaCDR3b noMHC	0,023	0,222	0,640	0,849	0	0	0,000	0,000
sasaCDR1a	-2,105	-1,884	0,000	0,000	1	1	**-2,105**	**-1,884**
sasaCDR1b	-0,543	-0,250	0,000	0,005	1	1	**-0,543**	**-0,250**
sasaCDR2a	-1,004	-1,606	0,000	0,000	1	1	**-1,004**	**-1,606**
sasaCDR2b	-1,001	-0,271	0,000	0,008	1	1	**-1,001**	**-0,271**
sasaCDR3a	-2,448	-1,661	0,000	0,000	1	1	**-2,448**	**-1,661**
sasaCDR3b	-2,616	-2,763	0,000	0,000	1	1	**-2,616**	**-2,763**
rmsfCDR1a	-0,031	-0,001	0,000	0,467	1	0	-0,031	0,000
rmsfCDR2a	-0,018	-0,006	0,000	0,365	1	0	-0,018	0,000
rmsfCDR3a	-0,047	-0,013	0,000	0,082	1	0	-0,047	0,000
rmsfCDR1b	-0,019	0,001	0,000	0,529	1	0	-0,019	0,000
rmsfCDR2b	-0,009	-0,011	0,003	0,231	1	0	-0,009	0,000
rmsfCDR3b	-0,020	-0,027	0,000	0,006	1	1	**-0,020**	**-0,027**
gyrABloop	-0,002	0,010	0,296	0,694	0	0	0,000	0,000
gyrVCAlinker	-0,003	0,006	0,096	0,877	0	0	0,000	0,000
gyrVCBlinker	0,005	0,004	0,965	0,652	0	0	0,000	0,000
					1	1		
sasaABloop	-0,003	0,543	0,454	1,000	0	1	0,000	0,543
sasaVCAlinker	-0,019	-0,433	0,293	0,001	0	1	0,000	-0,433
sasaVCBlinker	0,065	-0,096	0,889	0,101	0	0	0,000	0,000
rmsfABloop	-0,007	0,098	0,190	0,977	0	1	0,000	0,098
rmsfVC_Alinker	-0,001	-0,015	0,444	0,225	0	0	0,000	0,000
rmsfVC_Blinker	0,013	-0,006	0,995	0,387	1	0	0,013	0,000
n of H-bonds TCRa/b	-0,514	0,608	0,014	0,760	1	0	-0,514	0,000

Table S 3: Comparison of the results between the LC13 TCR and the A6 TCR. In the difference column the difference between the mean value of all TCRpMHC simulations versus the mean value all TCR simulation is shown (see methods). The first column shows this for the LC13 TCR and the second column for the A6 TCR. In order to evaluate which differences are more than random we performed a bootstrapping analysis separately for each descriptor and TCR. We assessed the percentile of the observed differences between TCRpMHC and TCR simulations within 1000 random permutations mixing TCRpMHC and TCR values. We consider only the highest 2.5% and the lowest 2.5% as relevant. On this basis we show in the rightest two columns only the difference values that pass either of these thresholds. Differences larger than 0 are highlighted in green while differences smaller than 0 are highlighted in red. The results for using the cohend and tvd instead of the pure difference were similar (data not shown).
